# Synthesis, Anticonvulsant, Antimicrobial and Analgesic activity of Novel 1,2,4-Dithiazoles

**DOI:** 10.4103/0250-474X.44614

**Published:** 2008

**Authors:** A. Gupta, P. Mishra, S. K. Kashaw, V. Jatav

**Affiliations:** Pharmaceutical Chemistry Division, Department of Pharmaceutical Sciences, Dr. H. S. Gour University, Sagar-470 003, India

**Keywords:** 1,2,4-Thiadiazoles, 1,2,4-dithiazole, antimicrobial, anticonvulsant, analgesic, neurotoxicity

## Abstract

A series of 1,2,4-dithiazole were synthesized from 1,2,4-thiadiazoles in the presence of CS_2_ and evaluated for their antimicrobial, anticonvulsant, analgesic and neurotoxicity potential. The compounds provided significant protection against maximal electroshock-induced seizures and seizures induced by 300 mg/kg of subcutaneous pentylenetetrazole administration. The designed compounds (3a-g) were screened *in vitro* for antibacterial activity against *Staphylococcus aureus Escherichia coli*, *Bacillus subtilis* and *Pseudomonas aeruginosa* and antifungal activity in fungal strains of *Candida albicans* and *Aspergillus niger.* Synthesized compounds exhibited moderate antibacterial and antifungal activity. *N,N* -Di-naphthalen-1-yl-*N* -(thioxo-5*H* -[1,2,4]dithiazol-3-yl)-guanidine and *N,N* -Bis-(4-fluoro-phenyl)-*N* -(5-thioxo-5*H* -[1,2,4]dithiazol-3-yl)-guanidine showed analgesic activity by tail flick method.

In recent years, several 1,2,4-dithiazole and their derivatives were found to have prominent pharmacological activities such as anticonvulsant, analgesic, antiinflammatory activity^1^–^3^. The present paper reports the synthesis and biological activity of some novel 1,2,4-Thiadiazoles and 1,2,4-dithiazoles. Aromatic/hetero aromatic amines were reacted with ammonium thiocyanate in the presence of concentrated HCl to give aryl thiourea (I). Comp I were oxidatively closed into 3-aryl amino-4-aryl-5-imino-D_2_ -1,2,4-thiadiazoline (II). Compound (II) on reacting with carbon disulfide gave the corresponding 3-thio-5-(N′-aryl-N′-arylguanyl)-1,2,4-dithiazoles. All the compounds showed satisfactory elemental analysis. IR and NMR spectra were consistent with the assigned structure (fig. 1).

**Fig. 1:**
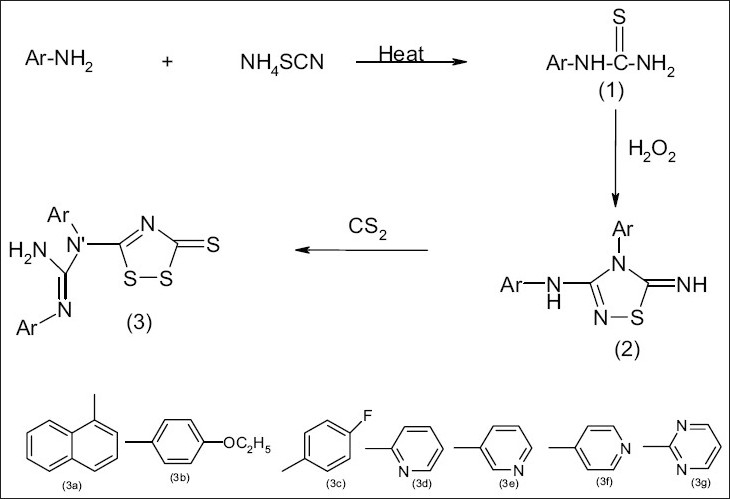
Scheme for the synthesis of 3-thio-5-(N′-aryl-N′-aryl guanyl)-1,2,4-dithiazole.

Melting points were determined by open capillary method by using Buchi 530 melting point apparatus and were uncorrected. The purity of the compounds was confirmed by thin layer chromatography using silica gel glass plates and a solvent system of benzene, ethanol (9:1). The spots were developed in iodine chamber and visualized under ultra violet lamp. All the compounds were subjected to elemental analysis (S and N) on Perkin Elemer-2400 instrument and the measured values agreed within ±0.4% with the calculated. ^13^C-NMR spectra were recorded on 13C Avance Bruker 300 MHz spectrophotometer. IR spectra in KBr disc were recorded on Testcan Shimadzu FTIR 8000. Mass spectra were obtained on Joel SX 102/M-6000 mass spectrometer applying FAB method.

Aryl thiourea was synthesized using a procedure in which aryl amine (0.1 mol) was taken in a 250-ml beaker containing 100 ml distilled water. Concentrated HCl (10 ml) was added and contents were warmed to dissolve the amine. Ammonium thiocynate (7.6 g, 0.1 mol) was added to the amine solution and the mixture was heated. The mixture was poured on crushed ice, the precipitate thus obtained was filtered off by suction and recrystallised from ethanol. Spectral analysis, m.p. and elemental analysis confirm the formation of the corresponding thiourea

3-amino-4-aryl-5-imino--1,2,4-thiadiazoline (2a-2g) were synthesized by warming corresponding aryl thiourea (1a-1g, 0.5 mol) in a conical flask and dissolving in warm 10 ml of HCl. Hydrogen peroxide (60-70 ml, 20 vol.) was added drop wise from the separating funnel with continuous stirring, the mixture was kept aside for 2 h. The oxidized mixture was diluted with water and separated sulphur was then removed by filtration. The filtrate on neutralization with dilute ammonia, gave precipitate which was collected by filtration and recrystallised from ethanol.

3-thio-5-(N′-aryl-N′-aryl guanyl)-1,2,4-dithiazole (3a-3g) were synthesized by dissolving corresponding thiadiazolines (2a-2g) (0.01 mol) in ethanol (20 ml). Carbon disulphide (12 ml) was added to it and the content was refluxed for 1h. The solution was cooled and thereafter yellow coloured crystals separate out. The products were recrystallised from ethanol. The physical properties of the title compounds are reported in Table 1.

**TABLE 1:** PHYSICAL AND SPECTRAL DATA OF THE COMPOUNDS (3a-g)

Code	% yield	mp°	Mol. formula	Elemental analysis (found%/ calculated%)	IR (KBr) cm^-1^/Molecular ion peak	^13^CNMR (δ ppm)
3a	70	130	C_23_H_16_N_4_S_3_	N(12.95/12.71)	3446 (NH), 1605 (C=N),	220 (C3), 163 (C5, C*),
				S(21.63/21.64)	1263 (C-N), 509 (S-S),	147.8 (C1’), 141.2 (C1”)
					1240 (C=S), 658 (C-S)/444	
3b	55	181	C_19_H_20_N_4_S_3_O_2_	N(12.94/12.94)	3334 (NH), 1635 (C=N),	220 (C3), 163 (C5, C*),
				S(22.20/22.19)	1305 (C-N), 512 (S-S),	140.6 (C1’), 132.9 (C1”)
					1246 (C=S), 672 (C-S)/432	
3c	66	172	C_15_H_10_N_4_S_3_F_2_	N(14.75/14.73)	3410 (NH), 1583 (C=N),	220 (C3), 163 (C5, C*),
				S(25.25/25.23)	1304 (C-N), 510 (S-S),	144.6 (C1’), 136.9 (C1”)
					1216 (C=S), 707 (C-S)/380	
3d	60	115	C_13_H_10_N_6_S_3_	N(24.24/24.24)	3428 (NH), 1624 (C=N),	220 (C3), 163 (C5, C*),
				S(27.60/27.70)	1325 (C-N), 512 (S-S),	156.5 (C2’), 147.7 (C2”)
					1240 (C=S), 723 (C-S)/346	
3e	64	118	C_13_H_10_N_6_S_3_	N(24.38/24.24)	3410 (NH), 1634 (C=N),	220 (C3), 163 (C5, C*),
				S(27.39/27.70)	1326 (C-N), 523 (S-S),	139(C3’), 145.1 (C3”)
					1279 (C=S), 676 (C-S)/346	
3f	71	126	C_13_H_10_N_6_S_3_	N(24.34/24.24)	3405 (NH), 1618 (C=N),	220 (C3), 163 (C5, C*),
				S(27.55/27.70)	1282 (C-N), 512 (S-S),	149 (C4’), 155.3 (C4”)
					1242 (C=S), 682 (C-S)/346	
3g	58	136	C_11_H_8_N_8_S_3_	N(32.23/32.16)	3404 (NH), 1647 (C=N),	220 (C3), 163 (C5, C*),
				S(27.33/27.60)	1293 (C-N), 522 (S-S),	163.2 (C2’), 169.3 (C2”)
					1256 (C=S), 657(C-S)/348	

Serial dilution method was used for determining minimum inhibitory concentration (MIC)^4^,^5^ of the synthesized compounds. Bacterial strain of *Staphylococcus aureus* (MTCC no. 96), *Pseudomonas aeruginosa* (MTCC no. 424), *Bacillus subtilis* (MTCC no. 619) and *Escherichia coli* (MTCC no. 40) and fungal strain of *Candida albicans* (MTCC no. 227) and *Aspergillus niger* (MTCC no.1344) were procured from Institute of Microbial Technology, Chandigarh and used in the present study^6^,^7^. Nutrient broth (beef extract 1 g, yeast extract 2 g, peptone 5 g, sodium chloride 5 g, distilled water q.s. 1000 ml) was used as growth medium for bacteria and Sabouraund’s medium (dextrose 40 g, peptone 10 g, distilled water q.s. 1000 ml) was used for growth of fungus^8^,^9^. Cook's procedure of serial dilution was used to determine the MIC. Dimethylformamide (DMF) was used as solvent. A blank test was conducted to check the antimicrobial activity of DMF. The results are in Table 2. The study was simultaneously performed for norfloxacin and clotrimazole as standard drug for antibacterial and antifungal activity respectively.

**TABLE 2:** ANTIBACTERIAL AND ANTIFUNGAL SCREENING OF 3-THIO-5-(N′-ARYL-N′-ARYLGUANYL)-1,2,4-DITHIAZOLES

Code.	*S. aureus*	*B. subtilis*	*P. aeruginosa*	*E. coli*	*C. albicans*	*A. niger*
3a	36**	68	34**	> 100	100	52*
3b	> 100	66	100	> 100	18***	>100
3c	100	16***	> 100	> 100	>100	96
3d	> 100	76	38**	> 100	52*	14***
3e	> 100	24**	26**	> 100	44**	56*
3f	100	68	> 100	66	36**	12***
3g	100	78	> 100	12***	54*	36**
Norfloxacin	4	16	10	8	-	-
Clotrimazole	-	-	-	-	6	12

Mic values are in μg/ml.

*** ≤ 20 μg/ml = highly active

** 21-40 μg/ml = moderately active

* ≥ 40 μg/ml = mild active

Initial anticonvulsant evaluation of the compounds was undertaken by the anticonvulsant drug development (ADD) program protocol at National Institute of Health, USA^10^,^11^. Male albino rats (90-110 g) of either sex maintained in standard conditions for temperature, relative humidity, light/day cycles and fed with normal diet and water *ad libitum*, were used. Maximal seizures were induced by application of an electrical current across the brain via corneal electrodes. The stimulus parameters were 50 mA, AC in a pulse of 60 Hz for 200 ms (0.2 s). The test compounds were suspended in 0.5% methyl cellulose/water. All the compounds were administered i.p. in doses of 30, 100 and 300 mg/kg to one to four animals. After 30 min and 4 h of drug administration electroshock was applied using corneal electrodes. Disappearance of the hind leg extensor component of convulsion was used as positive criteria.

The test compounds were administered i.p. to all animals in a group in doses of 30, 100 and 300 mg/kg. Thirty min after i.p. injection 60 mg/kg pentylenetetrazole was injected subcutaneously. Observations were taken for absence or presence of clonic convulsive seizures after 30 min and 4h of administration. The results are presented in Table 3

**TABLE 3:** ANTICONVULSANT, NEUROTOXICITY AND ANALGESIC ACTIVITY OF 3-THIO-5-(N′-ARYL-N′-ARYL GUANYL)-1,2,4-DITHIAZOLES

Code	MES		ScPTZ		Neurotoxicity		Latent period of tail flick response (Sec.) (Mean ± SEM)^a^	
				
	0.5h	4h	0.5h	4h	0.5h	4h	Control	Treated (after 30 min)
3a	300	300	--	--	100	--	7.60±0.25	10.52±0.85*
3b	100	300	--	--	300	--	4.82±0.38	6.9±1.38*
3c	300	--	300	--	100	--	5.50±0.21	9.69±0.25*
3d	300	--	--	--	100	--	6.86±0.92	7.6±0.23
3e	300	--	--	--	100	--	6.35±0.23	7.80±0.61
3f	300	--	--	--	100	--	6.66±0.41	8.39±0.38*
3g	--	--	--	--	100	--	4.86±0.45	5.96±1.3
Phenytoin	30	100	--	--	100	100		
Carbamazepine	30	--	100	--	100	300		
Acetyl salicylic acid (10 mg/Kg)							6.03±0.55	10.22±0.83*

(--)Indicates absence of activity at maximum dose administered.

a Mean of three experiments n=6

* p<0.5

Neurotoxicity studies were performed using the rotorod test^12^,^13^. Mice were grouped into groups of four animals each and trained to stay stable on an accelerating rotorod of diameter 3.2 cm revolving at the rate of 6 revolutions per min. Animals were administered 30, 100 and 300 mg/kg i.p. of the compounds. Thirty minutes after the injection the mice was placed individually on the rotating rod. Neurotoxicity was indicated by the inability of the animal to maintain equilibrium on the rod for at least one min in each of three trials (Table 3).

Analgesic activity was determined using hot wire ‘Techno’ anlgesiometer provided with an arrangement for circulation of cold water to avoid heating of the area surrounding the wire. Animals of either sex were grouped into groups of six animals each. The rats were then put into a rat holder individually with the tail protruding out of the hole. The tail was then kept on the hot wire of the instrument in such a way that it did not touch the wire. It is presumed that on feeling pain, the rat would withdraw its tail. After 30 minutes of the administration of synthesised compounds (10mg/kg.) the reaction time was determined. Acetyl salicylic acid (10 mg/kg i.p.) was taken as reference drug. The results are reported Table 3.

The anticonvulsant activities of the synthesised compounds were established after i.p. administration in two seizure models in mice viz., maximal electroshock-induced seizures (MES) and seizures induced by subcutaneous pentylenetetrazole administration (ScPTZ). The animals were dosed with 30, 100 and 300 mg/kg of the test compounds and examined at 0.5 and 4 h after the injections for anticonvulsant activity. The minimum dose whereby bioactivity was demonstrated in half or more of the mice is presented in Table 3 along with the activity data of phenytoin and carbamazepine. In this series 90% of the compounds tested were found to be active in MES screen, while only one compound 3c showed activity in ScPTZ screen. Hence the compound exhibits MES selectivity. All the compounds showed activity after 0.5 h hence onset of the action is rapid. Compound 3b was found to be moderately active showing activity at 100 mg/kg. Only one compound 3c showed broad spectrum of activity whereas compound 3a and 3b showed the prolong duration of action, showing activity at 300 mg/kg after 4 h. It was observed that compounds having napthyl group as substituents increases the potency of compounds. This may be attributed to bulkiness of the naphthyl ring which increases the hydrophobicity of the molecules thereby increasing the penetration power of the compounds across the blood brain barrier. All compounds showed neurotoxicity at some dose level. The compound 3b, showed neurotoxicity at higher dose level at 300 mg/kg. All remaining compounds were found to be neurotoxic at 100 mg/kg. None of the compound showed neurotoxicity at the end of 4 h. The compounds 3c and 3a showed significant analgesic activity (P< 0.01) in tail flick response test similar to the acetyl salicylic acid. Compound 3a and 3f of this series were also good analgesic effect with significant value P< 0.05 as compared to the standard drug acetyl salicylic acid. Other compounds of this series showed insignificant analgesic activity. Analgesic study was undertaken with a view to find whether the compounds showing good potency so far as anticonvulsant activity is concerned. It was observed that generally a good anticonvulsant had analgesic property too. Compound 3a was found to be moderately active against *S. aureus* and *P. aeruginosa*. Compound 3e was found to be moderately active against *B. subtilis* and *E. coli*. Compound 3d also showed moderate activity against *P. aeruginosa*. Compound 3b was found to be active against *C. albicans* whereas 3d, 3f were found to be active against *A. niger*. 3e and 3f were also found to be moderately active against *C. albicans* and compounds 3d and 3g were found to be also mild active against *C. albicans* and moderately active against *A. niger*.

## References

[1] Bohme H, Ahrens KH (1974). 5-Aaryl-3H-1,2,4-dithiazoles: A new class of highly potent fungicides. Arch Pharm.

[2] Pandeya SN, Kumar A, Singh BN, Mishra DN (1978). Synthesis and biological activity of isodithiobiurets, dithiobiurets, and dithiazoles. Pharm Res.

[3] MacDonald JW, McKinnon DM (1967). 1,2,4-Dithiazole-3-thiones and Derivatives. Can J Chem.

[4] Cook AM, Brown MR (1954). The relation between heat activation and colony formation for the spores of bacillus stearothermophilus. J Pharm Pharmacol.

[5] Davis BD, Dubelcco R, Eisen HN, Gimsberg HS (1980). Microbiology.

[6] (1992). Catalogue of MTCC Institute of Microbial Technology (IMTECH).

[7] Pelczar MJ, Chan EC, Kreig NR (1993). Microbiology.

[8] Collins CH, Lyne PM (1976). Microbiological Methods.

[9] Aneja KR (1996). Experiments in Microbiology, Plant Pathology, Tissue Culture and Mushroom Cultivation.

[10] Krall PL, Penry JK, White BG, Kupferburg HJ, Swinyard EA (1978). Antiepileptic drug development: II, anticonvulsant drug screening. Epilepsia.

[11] Conley JP, Kohn H (1987). Functionalized DL-Amino acid derivatives: Potent new agents for the treatment of epilepsy. J Med Chem.

[12] Kinnard WJ, Carr CJ (1957). A preliminary procedure for the evaluation of central nervous system depressants. J Pharmacol Exp Ther.

[13] Dunham MS, Miya TA (1957). A note on a simple apparatus for detecting neurological deficit in rats and mice. J Am Pharmac Assoc Sci Edit.

